# Efficacy of pharmacological and non-pharmacological therapy on pain intensity and disability of older people with chronic nonspecific low back pain: a protocol for a network meta-analysis

**DOI:** 10.1186/s13643-023-02369-0

**Published:** 2023-11-07

**Authors:** Letícia Soares Fonseca, Juliana Pereira Silva, Mateus Bastos Souza, Rodrigo de Oliveira Mascarenhas, Hytalo de Jesus Silva, Mariana Gabrich Moraes Campos, Leani Souza Máximo Pereira, Murilo Xavier Oliveira, Vinicius Cunha Oliveira

**Affiliations:** 1 Postgraduate Program in Rehabilitation and Functional Performance, Universidade Federal dos Vales do Jequitinhonha e Mucuri (UFVJM), Diamantina, Brazil; 2 Department of Physiotherapy, Universidade Federal dos Vales do Jequitinhonha e Mucuri (UFVJM), Diamantina, Brazil; 3 Postgraduate Program in Health Sciences, Universidade Federal dos Vales do Jequitinhonha e Mucuri, Diamantina, Brazil; 4 Postgraduate Program in Rehabilitation Sciences, Universidade Federal de Minas Gerais, Belo Horizonte, Brazil

**Keywords:** Low back pain, Network meta-analysis, Aged, Disability

## Abstract

**Background:**

The purpose of this network meta-analysis (NMA) is to investigate the efficacy of pharmacological and non-pharmacological therapy on pain intensity and disability of older people with chronic nonspecific low back pain, providing comprehensive evidence for an informed decision-making.

**Methods:**

We will perform a systematic search to identify randomized controlled trials of pharmacological and non-pharmacological interventions for older people with chronic nonspecific low back pain. MEDLINE, Cochrane Library, Embase, AMED, PsycINFO, and PEDro will be searched without language or date restrictions. Our primary outcomes are pain intensity and disability. Risk of bias will be assessed for all studies using the revised Cochrane risk-of-bias (RoB) tool 2.0. For each pairwise comparison between the different interventions, estimated mean differences and their 95% confidence intervals will be presented. Standard pairwise meta-analyses will be performed using random effects models in STATA version 16. The competing interventions will be ranked using the surface under the cumulative ranking curve (SUCRA) for the outcomes of interest at short and long terms. The confidence in the results from NMA will be assessed using the Confidence in Network Meta‐Analysis (CINeMA) framework.

**Discussion:**

This NMA compares efficacy of interventions for nonspecific chronic low back pain in older people. It will provide reliable evidence for patients, clinicians, stakeholders, and researchers in this field where competing therapies, many of extraordinarily little value, are commonly used in clinical practice.

**Systematic review registration:**

PROSPERO CRD42022312565

**Supplementary Information:**

The online version contains supplementary material available at 10.1186/s13643-023-02369-0.

## Background

Low back pain (LBP) is defined as pain below the costal margin and above the inferior gluteal folds with or without leg pain (sciatica) [1, 2], and specific causes of this health condition cannot be identified in most episodes of the condition [3–5]. LBP is considered a worldwide public health issue [6, 7]. It is expected that 70 to 85% of the general population will present an episode of LBP during their lifetime, including older people [8]. Epidemiological studies found that the high prevalence of LBP in older people [9–11] brings disability and increases costs in this population [11–13].

Evidence-based guidelines for the management of LBP have supported mainly psychological and physical therapies with less focus on pharmacological therapies [6, 14–16], but they have overlooked older people specificities that should be considered in the decision-making processes (e.g., chronicity of the condition, comorbidities, and polypharmacy). In this way, efficacy of pharmacological and non-pharmacological therapies should be clarified in this population, considering their context [17, 18].

Previous systematic review of randomized controlled trials (RCTs) with pairwise meta-analysis showed current low-quality evidence supporting exercise and trigger point acupuncture on disability in older people with nonspecific LBP at short term [19]. However, conventional pairwise meta-analysis is limited to comparing two interventions at a time and to previous trials that directly investigated these interventions [20], and they may not provide all the information required to make a clinical decision. One way to explore all the direct and indirect evidence available is the network meta-analysis (NMA) [21]. This method allows comparisons of interventions that have never been directly investigated. In addition, it provides more accurate estimates for pairs of comparisons where the evidence is limited to a small number of low-power studies [22]. The aim of this NMA is to investigate the efficacy of pharmacological and non-pharmacological therapy on pain intensity and disability in older people with chronic nonspecific LBP.

## Methods

This NMA of RCTs was prepared using the Preferred Reporting Items for Systematic Reviews and Meta-Analyses Protocol (PRISMA-P) [23] and Cochrane recommendations [20]. We have completed the PRISMA-P checklist (Supplementary file 1). We will use the PRISMA-NMA extension statement to structure the contents of the actual systematic review and NMA [24]. The protocol was registered with the Open Science Framework (https://doi.org/10.17605/OSF.IO/4W6R5) and registered to PROSPERO database on March 27, 2022 (CRD42022312565).

### Eligibility criteria

Summary of eligibility criteria for Population, Intervention, Comparison, Outcome and Study design (PICOS) is available in Table 1.

**Table 1:** Summary of PICOS eligibility criteria

	**Inclusion criteria**
**Population**	Older people (≥ 60 years) with chronic nonspecific low back pain
**Intervention**	Pharmacological and non-pharmacological interventions
**Comparison**	Pharmacological and non-pharmacological interventions, placebo, sham, no intervention, and waiting list
**Outcome**	Pain intensity and disability
**Study design**	Randomized controlled trials (RCTs)

### Population

#### Inclusion criteria

Trials that aimed to investigate people aged 60 years or over according to the World Health Organization [8], with chronic nonspecific LBP, will be included. Chronic nonspecific LBP is defined as pain symptoms of unknown causes with duration of at least 12 weeks [3–5].

#### Exclusion criteria

Trials that investigated participants with suspected or confirmed specific spinal conditions (i.e., fracture, cancer, inflammatory or infectious diseases of the spine, cauda equina syndrome/widespread neurological disorder, radiculopathy) were excluded [25].

Trials that investigated efficacy of surgical treatments and other invasive procedures (e.g., spinal injections, radiofrequency denervation, deep brain and spinal cord stimulation [26]. Invasive procedures are recommended for the lower back only as a next-line treatment in secondary or tertiary care or refractory low back pain where conservative primary care treatments have failed and are not recommended in any guideline when low back pain is chronic and nonspecific [25].

### Interventions

Pharmacological and non-pharmacological interventions were considered, and a previous search was performed to classify the intervention nodes. The classifications are differentiated mainly by mechanisms of action. If an intervention does not fulfill our anticipated node classification, it will be included in a new group, respecting the principle of transitivity.

### Pharmacological therapies

- Antidepressants (SNRI), e.g., duloxetine, desvenlafaxine, levomilnacipran, venlafaxine, and milnacipran
- Antidepressants (SSRI), e.g., fluoxetine, fluvoxamine, paroxetine, escitalopram, citalopram, sertraline, and vilazodone
- Antidepressants (tricyclic), e.g., amitriptyline, amoxapine, desipramine, imipramine, doxepin, clomipramine, trimipramine, protriptyline, imipramine, nortriptyline, doxepin, and nortriptyline
- Nonsteroidal anti-inflammatory drug (NSAIDS), e.g., ibuprofen, naproxen, sulindac, ketoprofen, tolmetin, etodolac, fenoprofen, diclofenac, flurbiprofen, piroxicam, ketorolac, indomethacin, meloxicam, nabumetone, oxaprozin mefenamic acid, diflunisal, and fenoprofen
- Opioids (strong), e.g., morphine, hydromorphone, oxycodone, fentanyl, methadone, buprenorphine, diamorphine, and tapentadol
- Opioids (weak), e.g., codeine, hydrocodone, tramadol, pentazocine, and tilidine
- Muscle relaxants (skeletal), e.g., flupirtin, orphenadrine, dantrolene, carisoprodol, tizanidine, incobotulinumtoxin A, cyclobenzaprine, metaxalone, baclofen, methocarbamol, and chlorzoxazone
- Muscle relaxants (benzodiazepines), e.g., diazepam, estazolam, quazepam, alprazolam, chlordiazepoxide, clorazepate, lorazepam, flurazepam, clonazepam, temazepam, and midazolam
- Antipyretic analgesic (paracetamol)
- Antiepileptic, analgesic, and anxiolytic (pregabalin and gabapentin)

### Non-pharmacological therapies

- Acupuncture
- Dry needling
- Aerobic (cardiorespiratory endurance) exercise
- Resistance exercise
- Flexibility exercise (stretching)
- Neuromotor exercise
- McKenzie therapy
- Mixed exercise
- Manual therapy: Includes high-velocity thrust techniques (manipulation, adjustment) and lower velocity oscillatory techniques (mobilization)
- Massage: Soft tissue massage and acupressure
- Mindfulness: Mindfulness and mindfulness-based stress reduction
- Patient education
- Psychological therapy: CBT, operant therapy, behavioral therapy, and self-regulatory therapy
- Ultrasound therapy
- Photobiomodulation
- Electrotherapy
- Auriculotherapy
- Balneotherapy or spa therapy
- Cryotherapy
- Heat therapy

The definitions of each intervention node are in the Supplementary file 2. We will not exclude RCTs based on frequency, duration, or intensity of the delivered intervention. This node-making process is being done *a priori*; however, modifications will be made to adjust for the lack of transitivity if necessary. Since transitivity analysis will be conducted before the computation of effect sizes, modifications in node definitions will not be biased by results of NMA.

### Comparator

Comparisons of interest are any pharmacological or non-pharmacological active intervention, no intervention, waiting list, placebo, or sham. In the NMA, placebo and sham will be combined as one node, and no intervention and waiting list will be combined as another node. The reason for grouping the controls into two different nodes is that the placebo and sham control for potential nonspecific effects (e.g., patient-therapist interaction and patient preferences) plus the natural history of the health condition, whereas no intervention and waiting list control only for the natural history of the condition [27].

In this review, usual care will not be considered because of the lack of consistency among trials that may potentially violate the assumption of transitivity for the NMA. If a trial names a control group as usual or standard care but clearly states that their participants did not receive any active intervention during the study, it will be included as “no intervention.”

### Outcomes

Pain intensity and disability will be considered if measured using a valid instrument.

#### Pain intensity

We will preferentially extract data measured with numerical rating scales (NRS). When NRS is not available, we will use data measured with other valid instruments for the population of interest [28] in the following order: visual analog scale (VAS), verbal rating scale (VRS), or Likert scales. Instruments that evaluate multidimensional aspects of pain (e.g., Brief Pain Inventory—BPI, short-form McGill Pain Questionnaire—SF/MPQ) will be considered only if it presents separate results for pain intensity measured with its NRS or VAS. In this review, assessments of multidimensional aspects or interference of pain will not be considered. Published trials commonly report multiple assessments of pain intensity (e.g., worst pain, average pain, pain in the last week). We will preferably extract data of average pain in the last 24 h at the moment and in the last week. The instrument used to assess pain intensity will be reported in the characteristics of the included trials [29].

#### Disability

We will extract disability associated with LBP measured using the Roland-Morris Disability Questionnaire (RMDQ) or the Oswestry Low Back Pain Disability Questionnaire (ODQ) [30–32].

### Study designs

RCTs investigating at least two different interventions of interest as defined above will be included. Randomization can be at the individual or group level, and both parallel group and crossover designs will be included. For crossover designs, only data from the first trial period will be extracted to eliminate any possibility of carryover effects.

### Search strategy

Search strategies will be conducted on the following: MEDLINE, Cochrane Library, Embase, AMED, PsycINFO, and PEDro without language or date restrictions. Descriptors will be related to “randomized controlled trials,” “nonspecific low back pain,” and “older people.” Detailed search strategy will be presented as Supplementary file 3. In addition, we will hand search identified systematic reviews published in the field for potentially relevant full texts.

### Study selection

After searching, the references will be exported to an EndNote^®^ file, and duplicates will be removed. Then, two independent reviewers (L. F. and J. S.) will screen titles and abstracts and will assess potential full texts. Those trials fulfilling our eligibility criteria will be included in the review. If necessary, authors will be contacted by email to clarify information. Three emails will be sent 7 days apart. If the authors do not answer, the study will be excluded, and the reasons will be reported in a flowchart. Between-reviewer discrepancies will be resolved by a third reviewer (M. S.).

### Data extraction

Two independent reviewers (L. F. and J. S.) will extract characteristics and outcome data from included trials, and discrepancies will be resolved by a third reviewer (M. S.). Extracted data will include study design (i.e., parallel group, crossover, or cluster RCT), source of participants, age, intervention details (e.g., type and class of treatment, intervention details, duration, dosage, delivery method), and outcome data (including assessment used, timing, missing data details).

For our outcomes of interest, we will extract from all groups at short and long terms: sample sizes, means, and standard deviations (SDs) or standard error, range score, interquartile range, and confidence interval. Short-term effects will be considered follow-ups up to 12 weeks after the baseline, and long-term effects will be considered follow-ups over 12 weeks after the baseline. If more than one timepoint is available within the same follow-up period, the one closer to the end of the intervention will be considered. Mean changes from baseline and their SDs will be extracted if post-intervention scores are not available.

When trials include two or more arms comprehending different doses, frequency, or intensity of the same intervention, we will combine outcome data following the Cochrane recommendations [20].

In trials where SDs are not available, they will be imputed from the standard error, confidence interval, *p*-value, range values, interquartile interval, or from other similar trials included, following the recommendations [20]. When the data is presented in graphs, we will extract using Ycasd software [33]. When imputations are not possible, the authors will be contacted by email. If the authors do not respond, the study will be included in the review, but it will be excluded from the quantitative analysis. Our primary interest is the effect of assignment to intervention, so we will consider results for the intention‐to‐treat (as randomized) population. If data are missing due to participant dropout, we will use reported results for participants that completed the study. Data extraction will be conducted by two independent reviewers (L. F. and J. S.) using previously prepared electronic forms. Discrepancies will be resolved by a third author (M. S.).

For crossover RCTs, we will only consider results from the first randomization period to avoid carryover effects. We do not expect to find any cluster RCT, but if available, we will include them, and data will be extracted following the recommendations in the Cochrane Handbook [20].

### Risk-of-bias assessment using the Cochrane risk-of-bias tool (RoB 2)

Two independent trained reviewers (L. F. and J. S.) will assess methodological quality of included trials. Risk of bias will be assessed for all trials using the revised Cochrane risk-of-bias (RoB) tool 2.0 [34] The following five domains will be assessed: (1) bias arising from the randomization process, (2) bias due to deviations from intended interventions, (3) bias due to missing outcome data, (4) bias in measurement of the outcome, and (5) bias in selection of the reported result. We will use the algorithms described in the instrument for classification of each domain as follows: (1) low risk of bias, (2) some concerns, and (3) high risk of bias. The judgment of the overall risk of bias of the included trial will follow the rule: (1) low risk of bias, low risk of bias for all domains; (2) some concerns, some concerns for at least one domain but no high risk of bias in any domain; and (3) high risk of bias, high risk of bias in at least one domain or have some concerns for multiple domains in a way that substantially lowers confidence in the result. The evaluation of the domain “bias due to deviations from the intended interventions” will be done with an interest in quantifying the effect of the attribution to the interventions in the baseline, regardless of whether the interventions are received as intended.

In multi-arm trials that contain more than two groups of interest, the risk of bias will be assessed for each possible comparison between pairs of interventions. For instance, in a trial that investigated interventions A, B, and C, the risk of bias will be assessed for the comparison of A and B, A and C, and B and C. Moreover, assessments will be made for each outcome and time point separately. A third reviewer (M. S.) will resolve between-reviewer discrepancies, and inter-rater reliability to use RoB tool 2.0 will be investigated using Kappa after reviewers training.

The results of this assessment will be used for downgrading the quality of the evidence using the Confidence in Network Meta‐Analysis (CINeMA) framework and for the sensitivity analysis (as described below).

### Strategy for data synthesis and analysis

We will provide a descriptive table summarizing the key characteristics of each eligible study, including interventions, patient populations, and trial characteristics. A network diagram will show which intervention classes were compared, with larger network nodes indicating a greater number of patients and thicker connecting lines between nodes indicating a greater number of trials.

For each pairwise comparison between the different interventions, we will present mean differences (MDs) for pain intensity and disability outcomes, both with its respective 95% confidence intervals (CIs). We will assess the presence of clinical heterogeneity within each pairwise comparison by comparing the trial and study population characteristics across all eligible trials.

Standard pairwise meta-analyses will be performed using a random effects model in STATA version 16 (StataCorp LLC, College Station, TX, USA) for all comparisons with at least two trials. Statistical heterogeneity within each pairwise comparison will be assessed using the *I*^2^ statistics, where an *I*^2^ ≥ 50% indicates heterogeneity [20].

Assumption of transitivity will be evaluated by comparing the distribution of study and population characteristics that could act as effect modifiers across the different pairwise comparisons. Random-effects NMA will be conducted if there is no evidence for important intransitivity. We will perform analyses using the network and network graphs packages in STATA.

In NMA, we will assume a common estimate for the heterogeneity variance across the different comparisons. The assumption of coherence will be evaluated statistically using both local and global approach. Specifically, side-splitting method will be used to evaluate incoherence for every comparison with available direct evidence and the design-by-treatment interaction model to tests incoherence in the entire network. In case of significant incoherence, its possible sources will be investigated by the assessment of an uneven distribution of effect modifiers across groups of trials that compare different interventions. Therefore, the distribution of clinical and methodological variables that we suspect may be potential sources of either heterogeneity or inconsistency in each comparison-specific group of trials will be investigated.

The competing interventions will be ranked using the surface under the cumulative ranking curve (SUCRA) for the outcomes of interest (i.e., pain intensity and disability) at short and long terms.

### Assessment of quality of evidence using Confidence in Network Meta‐Analysis (CINeMA)

The confidence in the results from NMA will be assessed using the CINeMA framework. CINeMA is broadly based on the GRADE framework, with several conceptual and semantic differences. It covers 6 domains: (i) within-study bias (referring to the impact of risk of bias in the included studies), (ii) reporting bias (referring to publication and other reporting bias), (iii) indirectness, (iv) imprecision, (v) heterogeneity, and (vi) incoherence. For each domain, three-level judgment can be performed (no concerns, some concerns, or major concerns) [35]. For imprecision, heterogeneity, and incoherence judgments, the equivalence range will be defined according to the minimal clinically important change (MCID) available for older people with LBP (i.e., two points on a 11-point NRS for pain intensity and five points in a 25-point RMDQ for disability) [36]. For the within-study bias domain, the weighted average of the risk of bias as calculated by CINeMA will be used. Two authors (L. F. and J. S.) will independently make the judgments about quality of the evidence (high, moderate, low, or very low), and discrepancies will be resolved by a third reviewer (M. S.).

## Discussion

This NMA will provide important results for clinical decision-making processes by providing a comparative assessment of various interventions for older people with chronic nonspecific LBP. It will provide reliable evidence for patients, clinicians, stakeholders, and researchers [37] in this field where competing therapies, many of extraordinarily little value, are commonly used in clinical practice.

One of the strengths of this NMA will be that the process of elaborating the intervention nodes is being defined *a prori* and based on previously published definitions in reviews, to make this step more transparent and based on clinical arguments [27]. The risk of bias of the included studies will be analyzed using the RoB 2.0 tool, for each comparison between trials and for each outcome and timepoint in each trial [34]. In addition, we will assess the confidence of the evidence that contributes to the estimation of interventions included through the CINeMA approach [35]. This NMA will be of great importance because it will contribute to a classification of pharmacological and non-pharmacological interventions according to their efficacy.

### Supplementary Information

**Additional file 1.** PRISMA-P 2015 checklist

**Additional file 2.** Definitions of each intervention node

**Additional file 3.** Search strategy

## Abbreviations

SUCRA: Surface under the cumulative ranking curve
CINeMA: Confidence in Network Meta‐Analysis
LBP: Low back pain
NMA: Network meta-analysis
RCTs: Randomized controlled trials
PRISMA-P: Preferred Reporting Items for Systematic Reviews and Meta-Analyses Protocol
PICOS: Population, Intervention, Comparison, Outcome and Study design
SNRIs: Serotonin and noradrenaline (norepinephrine) reuptake inhibitors
SSRIs: Selective serotonin reuptake inhibitors
NSAIDS: Nonsteroidal anti-inflammatory drug
