# Supplementary material for: Efficacy of pharmacological and non-pharmacological therapy on pain intensity and disability of older people with chronic nonspecific low back pain: a protocol for a network meta-analysis
Source: Syst Rev. 2023 Nov 7;12:205. doi: 10.1186/s13643-023-02369-0 (PMC10629194; doi:10.1186/s13643-023-02369-0)
Supplement: Supplementary file 2 — Additional file 2. Definitions of each intervention node [file 13643_2023_2369_MOESM2_ESM.docx]

**Additional file 2**. Definitions of each intervention node.

Intervention classes defined according to interventions commonly used in the treatment of low back pain. The definitions are based on previously published randomized controlled trials and systematic reviews that investigated the effectiveness of interventions in low back pain.

**Pharmacological treatments**

**1. Antidepressants (SNRI)**

Serotonin and noradrenaline (norepinephrine) reuptake inhibitors (SNRIs) act on noradrenergic and serotonergic neurons in the nervous system. Serotonin and noradrenaline are implicated in the mediation of endogenous pain inhibitory mechanisms. Treatment with SNRI increases transmission of these neurotransmitters and may improve disease states associated with serotonin and noradrenaline deficiencies such as pain, fatigue and cognitive deficits. (1) E.g. duloxetine, desvenlafaxine, levomilnacipran, venlafaxine, milnacipran. (2)

**2. Antidepressants (SSRI)**

Serotonin reuptake inhibitors (SSRIs) act on serotonergic neurons in the nervous system. Serotonin is implicated in the mediation of endogenous pain inhibitory mechanisms.Treatment with SSRI increases the functional expression of serotonin and may improve pain, fatigue, and cognitive deficits. (3) E.g. fluoxetine, fluvoxamine, paroxetine, escitalopram, citalopram, sertraline, vilazodone. (2)

**3. Antidepressants (tricyclic)**

Tricyclic antidepressants [TCAs] can produce an analgesic effect by disturbing norepinephrine and serotonin reuptake in the descending inhibitory system hence augment the amount of those neurotransmitters in the synaptic cleft . Each TCA has a different effect on monoamine reuptake. Amitriptyline, clomipramine, and imipramine are considered as dual-type TCA because they inhibit both serotonin and norepinephrine reuptake, whereas nortriptyline and desipramine are particularly inhibiting norepinephrine reuptake . Tricyclic antidepressants have a wide mechanism of action. They can interact with histamine, muscarinic, Nmethyl-D-aspartate [NMDA], and alpha-adrenergic receptors. (4) E.g. amitriptyline, amoxapine, desipramine, imipramine, doxepin, clomipramine, trimipramine, protriptyline, imipramine, nortriptyline, doxepin, nortriptyline. (2)

**4. Nonsteroidal anti-inflammatory drug (NSAIDS)**

NSAIDs inhibit the COX enzymes and can therefore inhibit the production of prostaglandins. Consequently this can reduce inflammation, pain and fever. COX‐1 produces prostaglandins that also support platelets and protect the stomach lining. It also helps to maintain kidney function. COX‐1 inhibition can raise the risk of renal insufficiency and gastro‐intestinal adverse events, such as gastritis or stomach bleeding.There are two types of NSAIDs: non‐selective NSAIDs, which inhibit both COX‐1 and COX‐2 enzymes, and selective NSAIDs, which inhibit only the COX‐2 enzyme. Both selective and non‐selective NSAIDs are available for pain treatment, and the choice of NSAID is mostly based on the different possible known adverse events, convenience of use, and cost. (5) E.g. Ibuprofen, naproxen, sulindac, ketoprofen, tolmetin, etodolac, fenoprofen, diclofenac, flurbiprofen, piroxicam, ketorolac, Indomethacin, meloxicam, nabumetone, oxaprozin mefenamic acid, diflunisal, fenoprofen. (2)

**5. Opioids**

Opioids are generally classified as either weak or strong. These terms refer to relative efficacy rather than potency; weak opioids exhibit a ceiling to their analgesic effect, limited principally by increased adverse reactions.

**5.1.** **Opioids (strong)**– e.g. morphine, hydromorphone, oxycodone, fentanyl, methadone, buprenorphine, diamorphine, tapentadol. (6, 7)

**5.2. Opioids (weak)-** e.g. codeine, hydrocodone, tramadol, pentazocine, tilidine. (6)

**6. Muscle relaxants (Skeletal)**

The non‐benzodiazepines, however, are structurally unrelated compounds that may indirectly relax skeletal muscle by blocking postsynaptic neurons in the spinal cord and the descending reticular formation in the brain. Baclofen is a gamma aminobutyric acid (GABA) derivative that inhibits neural transmission at the spinal level and also depresses the central nervous system. e.g. flupirtin, orphenadrine, dantrolene, carisoprodol, tizanidine, incobotulinumtoxinA, cyclobenzaprine, metaxalone, baclofen, methocarbamol, chlorzoxazone. (2)

**7. Muscle relaxants (benzodiazepines)**

The analgesic effects of benzodiazepines are predominantly mediated through activation of neuronal GABA A receptors. (8) e.g. diazepam, estazolam, quazepam, alprazolam, chlordiazepoxide, clorazepate, lorazepam, flurazepam, clonazepam, temazepam, midazolam. (2)

**8. Antipyretic analgesic (Paracetamol)**

Paracetamol has a spectrum of action similar to a weak NSAID. It inhibits COX-1 and COX-2 through metabolism by the peroxidase function of these isoenzymes. The mode of action of paracetamol is unclear. Its main effects appear to be exerted by interaction with neurotransmitters in the central nervous system, although it may act in part by inhibiting prostaglandin synthesis in peripheral tissues. (9)

**9. Antiepileptic, analgesic, and anxiolytic (Pregabalin)**

Pregabalin has a mechanism of action similar to gabapentin, binding to calcium channels and reducing calcium influx as well as influencing GABAergic neurotransmission.(10) This mode of action confers antiepileptic, analgesic, and anxiolytic effects. It is more potent than gabapentin due to a higher affinity for calcium channels and is therefore used at lower doses, with substantial differences in gastrointestinal absorption.(11) The dosing regimen for pregabalin is two times daily.

**Non-pharmacological treatments**

**10. Acupuncture**

Acupuncture will be defined as “use of traditional acupuncture theory, with needles being inserted into classic meridian points, extra points or ah-shi points.” It will be excluded studies in which the acupuncture treatment did not involve needling (acupressure or laser acupuncture) (FURLAN et al., 2005). (12)

**11. Dry needling**

Dry needling will be defined by applying needles to myofascial trigger points identified by palpation (FURLAN et al., 2005). (12)

**12. Aerobic (Cardiorrespiratory Endurance) Exercise**

Aerobic exercise will be defined according to the guidelines of the American College of Sports Medicine, which can also be called cardiorespiratory exercise. It can include a wide range of activities, such as walking, running, cycling and dancing, being performed at submaximal intensity and lasting from minutes to hours. (13)

**13. Resistance Exercise**

Resistance exercise will be defined as an intervention that aims to gain resistance, strength, muscle power or a combination of these. We will not establish a minimum duration of specific intervention. Resistance during training can be applied through free weights, elastic bands, weight machines, calisthenics (the use weight of a body segment or segments moving against gravity as the load for the exercise). (13)

**14. Flexibility Exercise (Stretching)**

Flexibility exercise will be defined as an exercise that aims to gain or maintain the range of motion of joint and muscle structures. (13)

**15. Neuromotor Exercise**

Neuromotor exercise training involves motor skills such as balance, coordination, gait, and agility, and proprioceptive training and is sometimes called functional fitness training. Other multifaceted physical activities sometimes considered to be neuromotor exercise involve varying combinations of neuromotor exercise, resistance exercise, and flexibility exercise and include physical activities such as tai ji (tai chi), qigong, and yoga. (13)

**17. McKenzie therapy**

Exercise training following traditional McKenzie principles such as repeated passive spine movements and sustained positions performed in specific directions. (14)

**18. Mixed exercise**

Mixed training will be defined as an intervention that contain at least two of the following modalities of exercise (i.e. aerobic, resistance and flexibility). Each type of exercise had to contribute as a significant part of the exercise intervention. Other types of exercise (e.g., co ‐ordination, balance, and relaxation involving voluntary muscle contractions), could also contribute to the intervention.(15)

**19. Manual therapy**

Includes high velocity thrust techniques (manipulation, adjustment) and lower velocity oscillatory techniques (mobilisation): Spinal manual therapy was defined as any manual technique that moves one or more joints within normal ranges of motion and aims at improving spinal joint motion or function, i.e., any mobilisation or spinal manipulation technique. (16)

**20. Massage**

Massage will be defined as any soft tissue manipulation done with the hands or with auxiliary devices. The massage may have been applied to any part of the body. Soft tissue massage, acupressure. (17)

**21. Mindfulness**

Being aware of the present moment in a non‐judgemental and accepting way. Mindfulness, mindfulness-based stress reduction. (18)

**22. Psychological therapy**

It will be defined as an intervention that aims to modify negative thoughts about pain and promote behavioral changes in order to improve function and develop coping strategies to deal with pain. Cognitive behavioural therapy, operant therapy, behavioural therapy, self-regulatory therapy. (19)

**22.1 Cognitive behavioural therapy:** Uni-disciplinary programmes including combined concepts: where it is one profession (usually Physio) who may be using cognitive - behavioural principles or a cognitive - behavioural approach, alongside exercise / education. (20)

**23.** **Patient education**

Back school (e.g. instruction on anatomy and function of the back), brief educational intervention, advice on importance of staying active, reassurance. (21)

**24. Ultrasound therapy**

Ultrasound therapy is the use of sound waves (vibrations) to treat medical problems. It is commonly used to treat low back pain. A healthcare provider rubs a hand‐held machine against the skin on the lower back. The machine produces vibrations that go through the skin. The aim is to deliver heat and energy to body parts under the skin, to reduce pain and speed up recovery. (22)

**25.** **Photobiomodulation**

Low level laser therapy (LLLT) is a non‐invasive light source treatment that generates a single wavelength of light. It emits no heat, sound, or vibration. It is also called photobiology or biostimulation. LLLT is believed to affect the function of connective tissue cells (fibroblasts), accelerate connective tissue repair and act as an anti‐inflammatory agent. Lasers with different wavelengths, varying from 632 to 904 nm, are used in the treatment of musculoskeletal disorders. (23)

**26. Electrotherapy**

Electrotherapy will be defined as a treatment that implements non-invasive electrical current to promote pain relief and improve function. This may include: transcutaneous electrical nerve stimulation (TENS), interferential current, galvanic current. Iontophoresis will not be considered because the medicinal ion component is supposed to have an additional effect to the electrical current. (24)

**27. Auriculotherapy**

Auriculotherapy (AT) is a complementary therapy, based on the idea that the ear is a microsystem which reflects the entire body, represented on the auricle, the outer portion of the ear. Mapping of the auricle has been described, according to the fact that pathology of different organs can induce specific changes in the auricle, for example color change or sensitivity. Treating specific areas, which somehow would be "connected" to the affected organ could also improve the functioning of the body or relieve pain. AT uses placement of needles at points tailored to the patient's pathology**.** (25)

**28. Balneotherapy**

Balneotherapy or spa‐therapy is an ancient and popular therapy. It involves spending time in an indoor pool filled with mineral water at temperature of between 31 to 34 degrees Celsius (88 to 93 degrees Farenheit). Different types of mineral water may be used in this therapy. (26)

**29. Cryotherapy**

Cryotherapy will be defined by applying cold, ice packs or massage with ice over painful areas or acupoints, with the objective of relieving pain. (27)

**30.** **Heat therapy**

Heat therapy will be defined through the application of hot packs, superficial heat, in order to decrease pain and/or stiffness, increase mobility, help to relax the muscles.(27)

**REFERENCES**

1. Welsch P, Üçeyler N, Klose P, Walitt B, W. H. Serotonin and noradrenaline reuptake inhibitors (SNRIs) for fibromyalgia. Cochrane Database of Systematic Reviews. 2018.

2. Chou R, Deyo R, Friedly J, Skelly A, Weimer M, Fu R, et al. Systemic Pharmacologic Therapies for Low Back Pain: A Systematic Review for an American College of Physicians Clinical Practice Guideline. Annals of internal medicine. 2017;166(7):480-92.

3. Walitt B, Urrútia G, Nishishinya MB, Cantrell SE, Häuser W. Selective serotonin reuptake inhibitors for fibromyalgia syndrome. The Cochrane database of systematic reviews. 2015;2015(6):Cd011735.

4. Leucht C, Huhn M, Leucht S. Amitriptyline versus placebo for major depressive disorder. The Cochrane database of systematic reviews. 2012;12:Cd009138.

5. Enthoven WT, Roelofs PD, Deyo RA, van Tulder MW, Koes BW. Non-steroidal anti-inflammatory drugs for chronic low back pain. The Cochrane database of systematic reviews. 2016;2(2):Cd012087.

6. Whittle SL, Richards BL, Husni E, Buchbinder R. Opioid therapy for treating rheumatoid arthritis pain. The Cochrane database of systematic reviews. 2011(11):Cd003113.

7. Chaparro LE, Furlan AD, Deshpande A, Mailis‐Gagnon A, Atlas S, Turk DC. Opioids compared to placebo or other treatments for chronic low‐back pain. Cochrane Database of Systematic Reviews. 2013(8).

8. Richards BL, Whittle SL, Buchbinder R. Antidepressants for pain management in rheumatoid arthritis. Cochrane Database of Systematic Reviews. 2011(11).

9. Maher C, Underwood M, Buchbinder R. Non-specific low back pain. Lancet (London, England). 2017;389(10070):736-47.

10. Taylor CP, Angelotti T, Fauman E. Pharmacology and mechanism of action of pregabalin: the calcium channel alpha2-delta (alpha2-delta) subunit as a target for antiepileptic drug discovery. Epilepsy research. 2007;73(2):137-50.

11. Bockbrader HN, Wesche D, Miller R, Chapel S, Janiczek N, Burger P. A comparison of the pharmacokinetics and pharmacodynamics of pregabalin and gabapentin. Clinical pharmacokinetics. 2010;49(10):661-9.

12. Furlan AD, van Tulder MW, Cherkin DC, Tsukayama H, Lao L, Koes BW, et al. Acupuncture and dry-needling for low back pain. The Cochrane database of systematic reviews. 2005(1):Cd001351.

13. Ferguson B. ACSM’s Guidelines for Exercise Testing and Prescription 9th Ed. 2014. J Can Chiropr Assoc. 2014;58(3):328-.

14. Owen PJ, Miller CT. Which specific modes of exercise training are most effective for treating low back pain? Network meta-analysis. 2020;54(21):1279-87.

15. Bidonde J, Busch AJ, Schachter CL, Webber SC, Musselman KE, Overend TJ, et al. Mixed exercise training for adults with fibromyalgia. The Cochrane database of systematic reviews. 2019;5(5):Cd013340.

16. Stochkendahl MJ, Kjaer P, Hartvigsen J, Kongsted A, Aaboe J, Andersen M, et al. National Clinical Guidelines for non-surgical treatment of patients with recent onset low back pain or lumbar radiculopathy. Eur Spine J. 2018;27(1):60-75.

17. Furlan AD, Giraldo M, Baskwill A, Irvin E, Imamura M. Massage for low-back pain. The Cochrane database of systematic reviews. 2015;2015(9):Cd001929.

18. Theadom A, Cropley M, Smith HE, Feigin VL, McPherson K. Mind and body therapy for fibromyalgia. The Cochrane database of systematic reviews. 2015;2015(4):Cd001980.

19. Roditi D, Robinson ME. The role of psychological interventions in the management of patients with chronic pain. Psychol Res Behav Manag. 2011;4:41-9.

20. National Guideline C. National Institute for Health and Care Excellence: Guidelines. Low Back Pain and Sciatica in Over 16s: Assessment and Management. London: National Institute for Health and Care Excellence (NICE)

Copyright © NICE, 2016.; 2016.

21. Engers AJ, Jellema P, Wensing M, van der Windt D, Grol R, van Tulder MW. Individual patient education for low back pain. Cochrane Database of Systematic Reviews. 2008(1).

22. Ebadi S, Henschke N, Forogh B, Nakhostin Ansari N, van Tulder MW, Babaei-Ghazani A, et al. Therapeutic ultrasound for chronic low back pain. The Cochrane database of systematic reviews. 2020;7(7):Cd009169.

23. Yousefi-Nooraie R, Schonstein E, Heidari K, Rashidian A, Pennick V, Akbari-Kamrani M, et al. Low level laser therapy for nonspecific low-back pain. The Cochrane database of systematic reviews. 2008(2):Cd005107.

24. Gibson W, Wand BM, Meads C, Catley MJ, O'Connell NE. Transcutaneous electrical nerve stimulation (TENS) for chronic pain - an overview of Cochrane Reviews. The Cochrane database of systematic reviews. 2019;4(4):CD011890-CD.

25. Suen LK, Wong TK, Chung JW, Yip VY. Auriculotherapy on low back pain in the elderly. Complement Ther Clin Pract. 2007;13(1):63-9.

26. Verhagen AP, Bierma‐Zeinstra SMA, Boers M, Cardoso JR, Lambeck J, de Bie R, et al. Balneotherapy for osteoarthritis. Cochrane Database of Systematic Reviews. 2007(4).

27. Brosseau L, Yonge KA, Robinson V, Marchand S, Judd M, Wells G, et al. Thermotherapy for treatment of osteoarthritis. The Cochrane database of systematic reviews. 2003;2003(4):Cd004522.
