# Supplementary material for: Efficacy of pharmacological and non-pharmacological therapy on pain intensity and disability of older people with chronic nonspecific low back pain: a protocol for a network meta-analysis
Source: Syst Rev. 2023 Nov 7;12:205. doi: 10.1186/s13643-023-02369-0 (PMC10629194; doi:10.1186/s13643-023-02369-0)
Supplement: Supplementary file 3 — Additional file 3. Search strategy [file 13643_2023_2369_MOESM3_ESM.docx]

**Additional file 3.** Search strategy.

OVID (MEDLINE, AMED, EMBASE, COCHRANE, PSYCINFO)

1. Randomized Controlled Trial/

2. Controlled Clinical Trial/

3. randomised controlled trial$.mp.

4. randomised controlled trial$.mp.

5. controlled clinical trial$.mp.

6. random allocation.mp. or Random Allocation/

7. Clinical Trial/

8. clinical trial$.mp.

9. Comparative Study/

10. cross-over studies.mp. or Cross-Over Studies/

11. Backache.mp. or Back Pain/

12. lumbago.mp. or Low Back Pain/

13. lumbar spine pain.mp.

14. (spin$ adj5 pain).mp.

15. aged.mp. [mp=ab, hw, kw, ti, ot, tx, ct, sh, tc, id, tm, tn, dm, mf, dv, fx, dq, nm, kf, px, rx, an, ui, sy]

16. aging.mp. [mp=ab, hw, kw, ti, ot, tx, ct, sh, tc, id, tm, tn, dm, mf, dv, fx, dq, nm, kf, px, rx, an, ui, sy]

17. older*.mp. [mp=ab, hw, kw, ti, ot, tx, ct, sh, tc, id, tm, tn, dm, mf, dv, fx, dq, nm, kf, px, rx, an, ui, sy]

18. elder*.mp. [mp=ab, hw, kw, ti, ot, tx, ct, sh, tc, id, tm, tn, dm, mf, dv, fx, dq, nm, kf, px, rx, an, ui, sy]

19. ancient*.mp. [mp=ab, hw, kw, ti, ot, tx, ct, sh, tc, id, tm, tn, dm, mf, dv, fx, dq, nm, kf, px, rx, an, ui, sy]

20. grey haired.mp. [mp=ab, hw, kw, ti, ot, tx, ct, sh, tc, id, tm, tn, dm, mf, dv, fx, dq, nm, kf, px, rx, an, ui, sy]

21. advanced in years.mp. [mp=ab, hw, kw, ti, ot, tx, ct, sh, tc, id, tm, tn, dm, mf, dv, fx, dq, nm, kf, px, rx, an, ui, sy]

22. 15 or 16 or 17 or 18 or 19 or 20 or 21

23. 1 or 2 or 3 or 4 or 5 or 6 or 7 or 8 or 9 or 10

24. 11 or 12 or 13 or 14

25. 22 and 23 and 24

PEDro

Abstract & Title: older* OR elder*

Therapy: not applicable

Problem: not applicable

Body Part: lumbar spine, sacro-iliac joint or pelvis

Subdiscipline: not applicable

Topic: not applicable

Method: clinical trial

Author/Association: not applicable

Title Only: not applicable

Source: not applicable

Published Since: not applicable

New records added since: not applicable

Score of at least: not applicable
